# Increased Sensitivity to Serotonin Syndrome in Cerebral Palsy

**DOI:** 10.1155/2022/5889506

**Published:** 2022-09-20

**Authors:** Adam Schindzielorz

**Affiliations:** Marshall Psychiatry, 1115 20th Street, Suite 205, 20th Street Professional Building, Huntington, WV 25703, USA

## Abstract

Serotonin syndrome is characterized by symptoms of neuromuscular and autonomic excitation and altered mental status. It is most often drug induced with antidepressants being the main precipitants. However, other classes have been implicated as well including antipsychotics, antiemetic and pain medications, and lithium. The syndrome is typically induced by the combination of two or more serotonergic agents; however, there have been instances of serotonin syndrome occurring while a patient is on a single medication. The literature is limited regarding the study of risk factors associated with the production of serotonin syndrome while on only monotherapy or otherwise atypically causative agents. One such risk factor may be underlying neuromuscular pathology. This study is the first case series to our knowledge reporting two separate cases of serotonin syndrome being induced in patients with cerebral palsy as an underlying common factor.

## 1. Introduction

The clinical ramifications of serotonin excess were first reported by Oats and Sjostrand in 1960, with the first attempt at standardizing diagnostic criteria occurring in 1991 by Sternbach et al. These criteria were further reviewed and honed into the more diagnostically sensitive and specific Hunter criteria by Dunkley et al. in 2003 [1, 2]. Serotonin syndrome is characterized by neuromuscular and autonomic excitation and altered mental status and typically occurs because of treatment with two or more known serotonergic agents [3]. Though the etiology of the illness has not yet been fully elucidated, serotonin receptors 5HT1a and 5HT2a, noradrenergic, gamma-aminobutyric acid, and dopamine pathways have been implicated [3, 4].

Unfortunately, the study of serotonin syndrome as it relates to neuromuscular disorders is limited. This represents a critical gap in the literature as this group demonstrates several clinical challenges in the management of serotonin toxicity. Our study focuses on two patients who experienced serotonin syndrome in the shared context of cerebral palsy, a motor disorder caused by nonprogressive injury to the developing brain that can alter both neuromuscular function and anatomic structure [5].

Importantly, this patient population has several factors salient to the development and treatment of serotonin syndrome. For example, patients with cerebral palsy have a higher incidence of depressive and anxiety-related illnesses when compared to the general population, potentially leading to higher risk of exposure to psychotropic medications. Additionally, 70–80% have spastic features that can make diagnosis of serotonin syndrome more difficult, which could lead to the delay in accurate identification and management [3, 6, 7]. We feel that our study adds to the literature by presenting the first known case series focusing on serotonin syndrome in cerebral palsy.

## 2. Case Series

Our first patient was an 18-year-old female with spastic cerebral palsy, diagnosed with an unspecified anxiety disorder for which fluoxetine was started at 20 mg daily. Three weeks after initiation, the patient developed symptoms of chest pain, shortness of breath, diaphoresis, disorientation, tremor, vomiting and diarrhea, and increased muscular tone requiring hospital admission.

On admission, her temperature was 99.5 degrees Fahrenheit with blood pressure of 158 mmHg/89 mmHg, heart rate of 102 beats per minute, and respirations of 18 breaths per minute with an oxygen saturation of 98% on room air. Admission labs showed white blood cell count of 12.8 k/cmm, c-reactive protein of 1.17 mg/dL, thyroid stimulating hormone of 3.670 mIU/mL, and free T4 of 1.07 ng/dL. Creatinine phosphokinase was not ordered. Psychiatry was consulted, and the patient was diagnosed with serotonin syndrome. She was started on diazepam 5 mg every six hours as needed for muscle rigidity and cyproheptadine 8 mg three times daily. There was marked improvement in spasticity, tremor, and autonomic signs over 24 hours. Prior to discharge, she received buccal swab genetic testing through GeneSight, which demonstrated increased activity of the CYP2D6 cytochrome p450 enzyme.

One month after discharge, she was reevaluated by an outpatient psychiatrist for continued symptoms of anxiety. She was started on sertraline, which was titrated to 50 mg daily. Roughly one month after initiation, her mother called the psychiatry provider to report reemergence of previous symptoms including diaphoresis, gastrointestinal distress, hypertension, and subjective increase in muscular tone. Sertraline was discontinued, and the patient was again placed on an outpatient cyproheptadine taper, which resulted in full resolution of her symptoms over a two-week period.

Our second patient was a 42-year-old female, also with spastic cerebral palsy, with a primary psychiatric diagnosis of bipolar I disorder. She was admitted to a local psychiatric facility for psychosis in the context of an acute manic episode. On admission, she was continued on home medications: gabapentin, tizanidine, clonazepam, and aripiprazole. Aripiprazole was titrated from 10 mg to 20 mg and later changed to a long-acting injectable formulation. Because no benefit was observed with higher dosing of aripiprazole, olanzapine was started and titrated to a total of 20 mg nightly. Lithium was also added for adjunctive mood stabilization. Several days following lithium initiation, the patient began to refuse oral intake, prompting lithium to be discontinued. After two more days, the patient began to physically decompensate, requiring a wheelchair for mobility. Shortly thereafter, she was found in her hospital room, unresponsive and hypoxic resulting in transfer to a local emergency department.

In the emergency department, she was diagnosed with acute respiratory failure in the context of suspected sepsis. Initial labs demonstrated lactic acid of 3.94 mmol/L, creatinine of 5 mg/dL, creatinine phosphokinase of 3160, and white blood cell count of 17.6 k/cmm. Urinalysis was negative for urinary tract infection. Of note, the lithium level was 1.2 despite having stopped the medication at least 48 hours prior. Admission vitals included temperature of 98.0 degrees Fahrenheit, blood pressure of 56 mmHg/37 mmHg, heart rate of 118 beats per minute, respiratory rate of 14 breaths per minute, and oxygen saturation of 92% on room air. All psychotropic medications were held, and she was admitted to the intensive care unit to be placed on mechanical ventilation. Vancomycin and piperacillin-tazobactam were started empirically. Computed tomography of the brain was performed without contrast and demonstrated a chronic right lateral ventricular dilatation without additional acute findings.

Neurology and psychiatry services were consulted roughly four days after admission due to concerns for possible neuroleptic malignant syndrome. On exam, the patient exhibited fever with temperature of 102.7 degrees Fahrenheit, hyperreflexia, skin flushing, inducible and spontaneous myoclonus, and tremor. She was diagnosed with serotonin syndrome by the psychiatry team. Because no infectious etiology was identified on lumbar puncture, blood, or fungal cultures, antibiotics were stopped and cyproheptadine started at 8 mg three times daily. The patient was aggressively hydrated and home tizanidine restarted at 2 mg nightly for muscular rigidity.

Over the course of five days, physical symptoms and vital signs improved. Cyproheptadine was tapered slowly, and quetiapine and oxcarbazepine started for mood stabilization in preparation for discharge home. However, once cyproheptadine was discontinued, inducible clonus, tremor, and autonomic instability reemerged. Quetiapine, oxcarbazepine, and tizanidine were discontinued, and cyproheptadine resumed at 8 mg three times daily which resulted in marked improvement of the patient's symptoms over an additional five days. Once symptoms had sufficiently resolved, she was restarted on quetiapine and oxcarbazepine and, having demonstrated continued stability, was discharged home with a slow cyproheptadine taper.

## 3. Discussion

Serotonin syndrome was diagnosed in both of our patients by using the Hunter criteria. To fulfill the Hunter criteria, a patient must have the presence of a serotonergic agent and meet one of the following: spontaneous clonus, inducible clonus plus agitation or diaphoresis, ocular clonus plus agitation or diaphoresis, tremor plus hyperreflexia, or hypertonia plus temperature above 38 degrees Celsius (100.4 Fahrenheit) plus ocular clonus or inducible clonus. Neuroleptic malignant syndrome was considered in our second case but was ruled out to the lack of hallmark signs such as severe rigidity or hyperpyrexia. Symptoms such as the presence of hyperreflexia and myoclonus were felt to be more consistent with serotonin syndrome, and the response to cyproheptadine felt to be confirmatory, as neuroleptic malignant syndrome would not be expected to receive therapeutic benefit from this medication.

Serotonin syndrome typically occurs in the context of treatment with two or more agents with at least one having direct serotonergic effects. The fact that symptoms developed in our patients with either selective serotonin reuptake inhibitor (SSRI) monotherapy or treatment with a lithium and olanzapine combination, both of which have indirect serotonergic action, is atypical [8].

Though we cannot rule out other potential influencing factors, a review of medication lists at the time of diagnosis did not reveal other agents that would have otherwise altered metabolism or potentiation of the medications that were felt to be causative. Furthermore, evaluation of cytochrome p450 metabolism via GeneSight genetic testing in our first patient revealed elevated CYP2D6 enzyme activity which would be expected to increase the metabolism of the prescribed SSRIs and thereby lower the likelihood for possible adverse effects. As such, given that both patients developed serotonin syndrome through atypical means in the context of an underlying diagnosis of cerebral palsy, the question is raised as to whether this group may be at higher risk for the development of serotonin syndrome.

The study of serotonin syndrome in patients with cerebral palsy is extremely limited. To our knowledge, the only other study directly addressing adverse events related to serotonergic agents in this patient population is a case series by Rone et al. In this study, two patients with cerebral palsy are reported to have developed clinically significant increases in muscle tone after being treated with SSRI monotherapy. The authors described the symptoms as similar to extrapyramidal side effects, more expected of antipsychotic medications, and speculated that dopaminergic pathways may have been involved [9].

Currently, there is no research regarding the possibility for increase sensitization of patients with cerebral palsy to serotonergic medications. However, the literature has identified serotonin's regulatory role on muscular tone via neuronal tracts in the pons and medulla [3]. Other studies have also evaluated muscular composition in cerebral palsy patients and have found aberrant muscular sarcomere and fiber composition with speculation that there may also exist pathologic changes in muscle innervation [5, 10]. Taken together, conditions appear to exist in which neuromuscular sensitivity to serotonin may be present in this population.

## 4. Conclusion

Our case series adds to the literature by being the first study to our knowledge to examine serotonin syndrome as it relates to cerebral palsy. We feel that the relationship between serotonin toxicity and cerebral palsy is important because of the diagnostic and management challenges that can be involved in this group. Ultimately, our study highlights the need for more research in this area as little information currently exists on this subject.

## Data Availability

No additional data were used to support this study.
